# Case report: Migraine that persisted for over 20 years disappears after treatment for pulmonary arteriovenous fistula

**DOI:** 10.1002/ccr3.3037

**Published:** 2020-06-23

**Authors:** Kazuyuki Kakeshita, Taro Yoneda, Hayato Koba, Kota Tanimura, Tsukasa Ueda, Tomoya Kaneda, Johsuke Hara, Kazuo Kasahara

**Affiliations:** ^1^ Department of Internal Medicine Komatsu Municipal Hospital Ishikawa Japan; ^2^ Department of Respiratory Medicine Kanazawa University Hospital Ishikawa Japan

**Keywords:** cerebral infarction, migraine, pulmonary arteriovenous fistula, shunt, transesophageal echocardiography

## Abstract

We presented a rare case of pulmonary arteriovenous fistula in a patient who suffered from migraine with optic aura for longer than 20 years. This case suggests that the migraine could be expected to disappear after treatment for pulmonary arteriovenous fistula.

## Graphical abstract

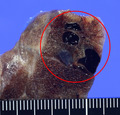

## 1 INTRODUCTION

Pulmonary arteriovenous fistula (PAVF) is an important disease state that is considered a risk factor of cerebral infarction.^1^ A high incidence (38.5%) of migraine with visual aura concomitant with PAVF has been reported.^2^ However, a case of migraine, which had persisted for more than 20 years, disappearing directly following PAVF treatment has not yet been reported. We experienced a case where this phenomenon occurred.

Here, we describe the clinical course of a patient with cerebral infarction resulting from PAVF; our examination suggested that this patient's migraine of unknown cause disappeared upon treating the PAVF.

## 2 CASE REPORT

**Figure 1:**
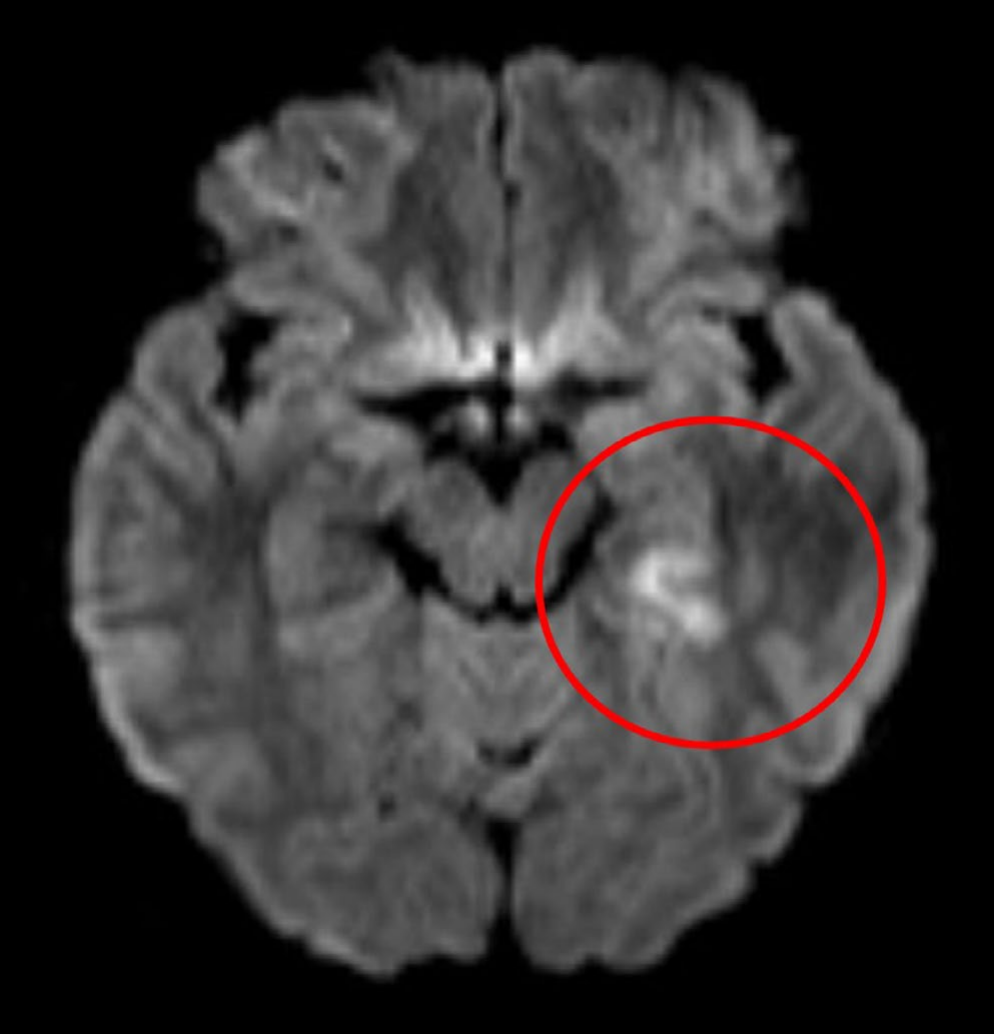
Diffusion‐weighted imaging of brain magnetic resonance imaging showed high signal in left backward lobus temporalis, which indicated fresh cerebral infarction (inside red circle)

A 41‐year‐old woman presented to our hospital in 2017. She has a history of migraine with visual aura once or twice every month for more than 20 years and recurrent cerebral infarction between the ages of 32‐37. At that time, it was thought that the cerebral infarction derived from migraine. This time, she lost right visual field after urinating upon waking and had loss of muscle strength in her right upper and lower extremities. Her visual field loss continued for 5 minutes accompanied with left occipital pain. Because the loss of muscle strength of her right upper and lower extremities persisted, she presented to the emergency department of our hospital. She had a clear sensorium and normal respiratory sound, with percutaneous oxygen saturation at 97%. Diffusion‐weighted brain magnetic resonance imaging (MRI) showed high signal in the left backward lobus temporalis, which indicated fresh cerebral infarction (Figure 1). In the past, she had magnetic resonance angiography (MRA) which had never shown vascular abnormality.

Repeated MRI also showed no abnormality. Upon admission, her right upper and lower extremities had loss of muscle strength with level 4 rating on manual muscle test, but returned to normal on the same day, and she had no sensory impairment. Additional blood examination showed no evidence of angitis and antiphospholipid antibody syndrome (APS). Examination of cerebrospinal fluid was performed, and the results were negative for the possibility of demyelinating disorder, such as meningitis (Table 1). Nevertheless, chest X‐ray showed a 1‐cm node in the left lower lung.

**Table 1:** Laboratory findings upon admission

**Blood**				**Blood gas analysis (room air)**	
WBC	7900 /μL	PIC	0.5 µg/mL	pH	7.402
Neut	50.4%	TAT	2.6 ng/mL	pO_2_	98.3 Torr
Eos	3.2%	Plasminogen	114%	pCO_2_	37.5 Torr
Lym	36.9%	Protein C	128%	HCO_3_^‐^	22.9 mEq/L
RBC	432 × 10^4^ /μL	Protein S	88%	SaO_2_	97.5%
Hb	13.2 g/dL	Lupus anticoagulant	Negative	A‐aDO_2_	9.8 Torr
MCV	91.1%	Anticardiolipin antibody	8 U/mL	**Cerebrospinal fluid examination**	
Plt	29.5 × 10^4 ^/μL	IgG	1057 mg/dL	Initial pressure	11 cmH_2_O
Na	137 mEq/L	IgA	171 mg/dL	Appearance	
CL	102 mEq/L	IgM	121 mg/dL	Colorless and transparent	
K	3.8 mEq/L	IgE	18.8 U/mL	Specific gravity	1.005
AST	26 U/L	C3	110 mg/dL	pH	7.6
ALT	33 U/L	C4	21.7 mg/dL	Sugar	66.0 mg/dL
TP	6.6 g/dL	RF	15 U/mL	Cell number	Mononuclear 1 /μL
Alb	4.0 g/dL	Antinuclear antibody	<40		Polynuclear 1 /μL
LDH	141 U/L	Anti SS‐A antibody	<1.0 U/mL	Protein	18 mg/d
BUN	15.4 U/L	Anti SS‐B antibody	1.1 U/mL	CL	124 mEq/L
Cr	0.41 mg/dL	PR3‐ANCA	<1.0 U/mL	Myelin basic protein	40.0 pg/mL
CRP	0.08 mg/dL	MPO‐ANCA	<1.0 U/mL	Oligoclonal band	Negative
BS	107 mg/dL	Cryoglobulin	Negative	IgG index	0.51
PT	99.00%				
PT‐INR	1.01				
APTT	31.40 s				
Fib	212 mg/dL				
D‐dimer	1.0 µg/mL				

**Figure 2:**
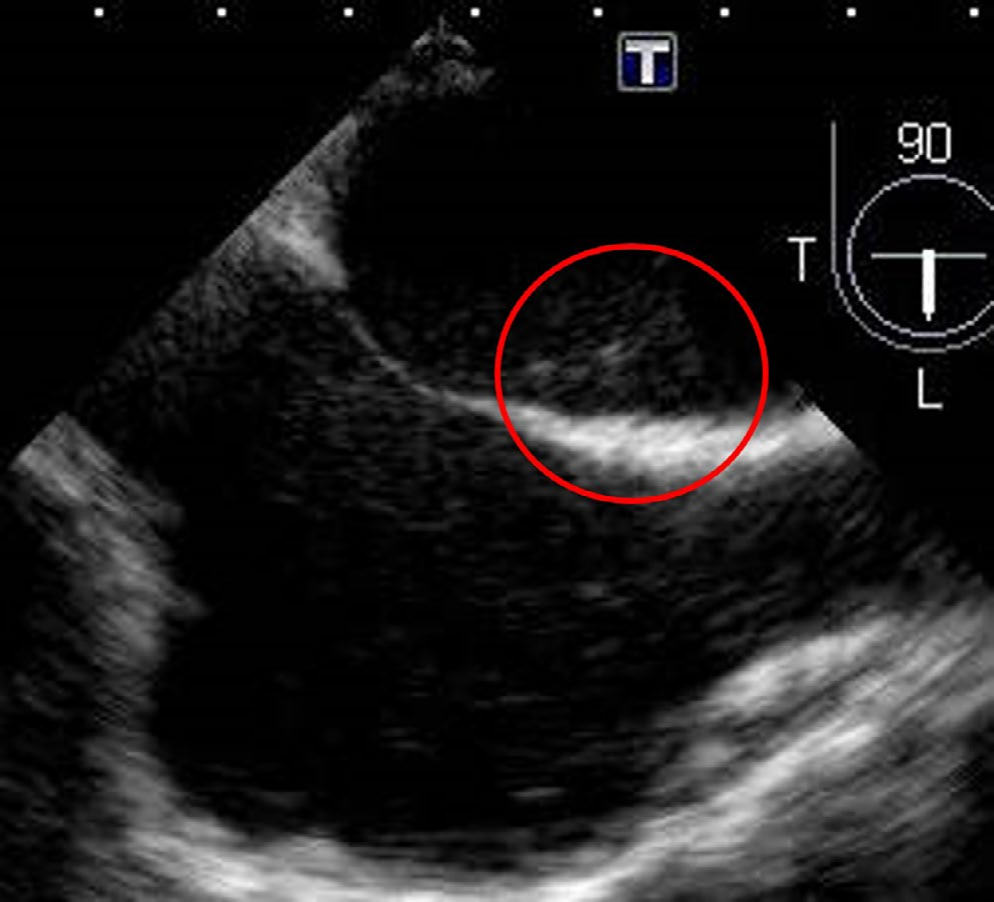
On transesophageal echocardiography, bubble contrast method showed time lag of the bubble about one or two breaths between the right and left atria

**Figure 3:**
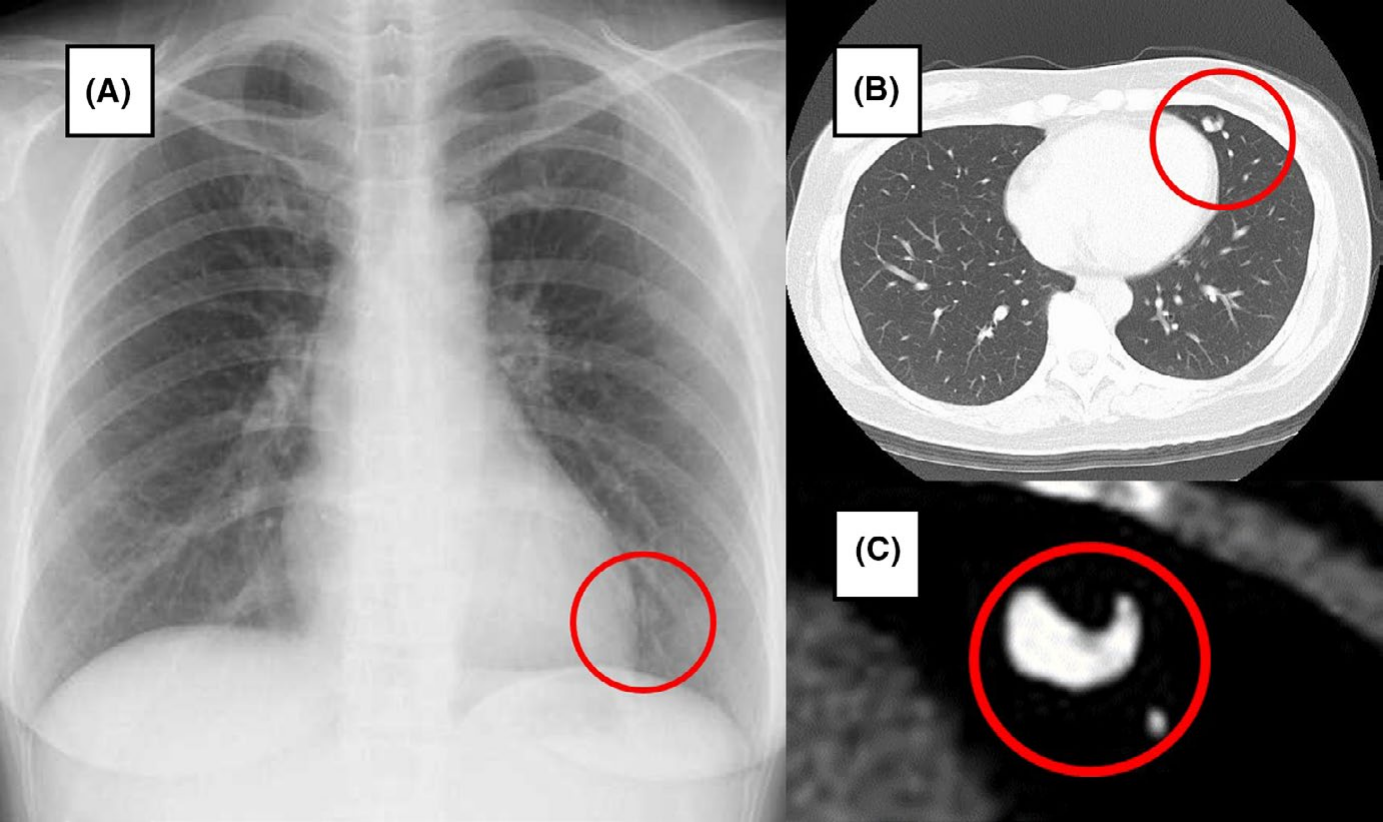
Chest X‐ray (A) and the enhanced computed tomography (B,C) showed pulmonary AVF which was 1.5 cm in the peripheral left superior lingular segment which was contrasted (C, inside red circle)

**Figure 4:**
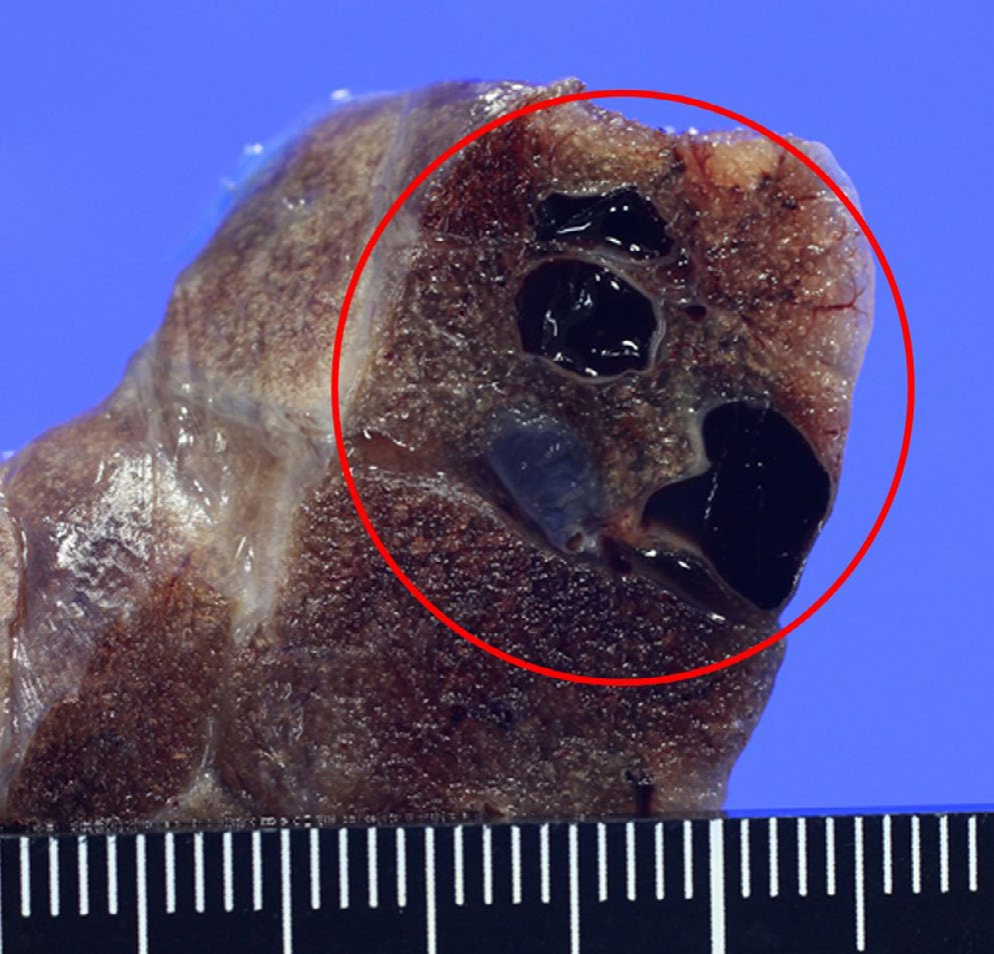
Resected specimen of pulmonary AVF which was 17 mm long formed by aggregation of the dilated vessels

To search for cause of the cerebral infarction, transesophageal echocardiography (TEE) was performed. Bubble contrast method showed no interatrial access. Nevertheless, there was a bubble lag time of about 1 or 2 breaths between the right and left atria (Figure 2), which indicated an existence of shunt in the atrium. Because PAVF was strongly suspected, an enhanced chest computed tomography (CT) was performed and showed PAVF which was 1.5 cm in the peripheral left superior lingular segment (Figure 3). Visible vessel of gastric mucosa was not confirmed with upper gastrointestinal endoscopy. The patient was not prone to nosebleed and there was no family history of hereditary telangiectasia, which can be diagnosed based on the presence of 4 criteria, known as the “Curaçao criteria”.^3^ As previously mentioned, we thought that this cerebral infarction had been derived from idiopathic PAVF as paradoxical embolism. Thus, left upper lobe partial resection was performed to treat PAVF. Macroscopically, we found blood blister‐like dilated vessels on the visceral pleura surface outside the left superior lingular segment, and a 17‐mm PAVF formed by aggregation of the dilated vessels was confirmed in the peripheral superior lingular segment (Figure 4). On postoperative day 33, transesophageal echocardiography was performed once again, and the shunt disappeared. After discharge from the hospital, chest CT did not show new PAVF. At the time this happened, she had been visiting a doctor regularly for 2 years. Her migraine, which would develop 1 or 2 times every month before the treatment of PAVF, completely stopped. It was thought that her migraine with optic aura which persisted for over 20 years derived from PAVF.

## 3 DISCUSSION

Pulmonary arteriovenous fistula is congenital in more than 90% of cases. Approximately 50% of congenital PAVF in the United States and 15% of congenital PAVF in Japan were complicated with hereditary telangiectasia,^4^ and 15% of hereditary telangiectasia was complicated with pulmonary AVF. In other words, when we encounter the diagnosis of PAVF, we should also take into consideration hereditary telangiectasia. It is reported that 9%–41% of central neurologic disease, such as cerebral stroke and brain abscess, was due to PAVF. In Japan, 0.5% of patients with recurrent cerebral infarction (4 of 754 cases) were reported to be caused by PAVF.^1^ Six years after the diagnosis of PAVF, the fatality rate was reported to be 11%. Therefore, aggressive management should be taken into consideration. Cases that present with hypoxemia, cardiac failure, central nervous system manifestation, and inflow artery diameter over 2–3 mm indicate treatment for PAVF,^5^ which includes surgical resection, such as segmental resection, excision of fistula, or transcatheter embolization. Surgical resection was performed for giant fistula or artery with large diameter of inflow in which blood current would stick. Currently, transcatheter embolization has become mainstream because of low invasiveness, low complication, and the ability to be performed with another embolization.^6^ It is reported that transcatheter embolization could be considered in the prevention of relapse of cerebral infarction caused by PAVF.^1^ Transcatheter embolization less invasively achieves 98% of treatment of PAVF and improves hypoxemia and hypoxemia. Nevertheless, it was reported that 1%–10% of cases with transcatheter embolization were recanalized in the long term.^5^ On the other hand, surgical resection was reported as definitive therapy whose outcome is unassociated with size of pathologic lesion.

Minimally invasive thoracoscopic surgery has evolved in recent years to provide reliable and definitive repair in patients who can be givengeneral anesthetic.^7^

In our case, surgical resection was chosen by the patient, because she was young and hoped for definitive repair. PAVF is an important disease state considered a risk factor of cerebral infarction as mentioned previously. It is noteworthy in this case that migraine was reported to coexist with PAVF and patent foramen ovale. Migraine with visual aura is recognized to coexist with PAVF in 38.5% of cases.^2^ In addition, it has been reported that migraine stopped after treatment of PAVF and patent foramen ovale.^8^, ^9^ Furthermore, the rate of migraine improvement was over 90% after 3 months of patent foramen ovale closure in patients with large‐diameter patent foramen ovale complicated by migraine.^10^

Nevertheless, prospective tests showed no remarkable differences in improvement of migraine.^11^, ^12^, ^13^ It was reported that migraine would stop after transcatheter closure of atrial septal defect.^14^ The mechanism of migraine improvement due to treatment of PAVF was not accurately known. Migraine with shunt is common in PAVF, patent foramen ovale, and atrial septal defect. It has been reported that migraine may occur from the inflow of serotonin and endothelin from venous system into arterial system via shunt.^15^ This same mechanism could be at work in pulmonary PAVF.

Transesophageal echocardiography is useful for detecting left atrial appendage thrombus, and left and right shunt when compared with transthoracic echocardiography.

Transesophageal echocardiography has been a prominent in the search for the cause of cerebral infarction.^16^ Likewise, in our case, TEE was an important examination.

In conclusion, in our case, PAVF was found when we searched for the cause of recurrent cerebral infarction in a patient who suffered from migraine with visual aura for more than 20 years. With the treatment of PAVF to prevent recurrent cerebral infarction, the sustained migraine stopped and disappeared.

While the cause of migraine with visual aura remains to be identified, we should take into consideration PAVF as cause of the migraine. Transthoracic echocardiography is very useful to detect shunt disease; however, in this case we used TEE. Once the diagnosis of PAVF is made in a patient with migraine, the migraine could be expected to disappear after treatment of PAVF.

## CONFLICT OF INTEREST

None declared.

## AUTHOR CONTRIBUTIONS

KK, HK, KT, and TU: drafted the contribution. TY: drafted, reviewed, edited, and conceived the contribution. TK, JH, and KK: conceived the contribution.
